# Structural modeling to understand the relationship among food safety knowledge, attitude, and self-reported HACCP practices in restaurant employees in Bangladesh

**DOI:** 10.1371/journal.pgph.0000103

**Published:** 2022-05-06

**Authors:** Md. Jahid Hasan, Rayhan Uddin, G. M. Rabiul Islam

**Affiliations:** Department of Food Engineering and Tea Technology, Shahjalal University of Science and Technology, Sylhet, Bangladesh; Universite de Montreal, CANADA

## Abstract

In this materialistic era, a substantial number of people are likely to have their meals outside of their homes and largely depend on the restaurants’ made food due to the prolonged working hours and tremendous pressure in workplace. Consequently, consumers expose themselves to risk and become vulnerable to illness caused by food. Unsafe food preparation and handling by restaurants’ workers have made food safety concern for public health. The study was aimed to examine the relationships among food safety knowledge, attitude, and Hazard Analysis Critical Control Point (HACCP) practices in restaurant employees in Bangladesh. A total of 360 employees from 120 restaurants participated in a face-to-face interview through a structured questionnaire comprising four sections: demographic characteristics, food safety knowledge, attitudes and practices. The mean scores for each survey item were calculated and used in Structural Equation Modeling (SEM), designed to assess interrelationships among the three sections related to food safety. Participants obtained a correct average score of 53.32% in food safety knowledge, with the highest and lowest correct scores in Good Hygiene Practices (GHPs) and HACCP practices, respectively. The highest score in the attitude section belonged to “self-improvement”, followed by “food safety concern”. A negative correlation was observed between knowledge with practices, knowledge with attitudes, and a positive correlation was observed between practices with attitudes. A significant positive correlation was observed between HACCP practices with shelf improvement (r = 0.54, *p* < 0.05) and the knowledge toward food poisoning with GHP practices (r = 0.55, *p* < 0.05). Self-improvement and food safety concerns are negatively correlated with food poisoning, GHP, and HACCP practice. This study demonstrated that restaurant employees in Bangladesh often lack knowledge regarding food safety and HACCP. So, in order to improve knowledge and attitude on safe HACCP practice among the restaurants employees’, proper education and interactive training sessions can be conducted.

## Introduction

Nowadays, food safety is a major concern worldwide, and millions of people become sick, while hundreds of thousands die every year because they consume unsafe food [1]. More than one-third of the total population in developing countries is affected by foodborne illnesses every year [2]. A recent estimate has reported that about 30 million people in Bangladesh suffer from foodborne diseases each year [3]. The burden of foodborne diseases in Bangladesh is increasing and occurs primarily due to food contamination in restaurants from unhygienic practices [4–6].

**Fig 1:**
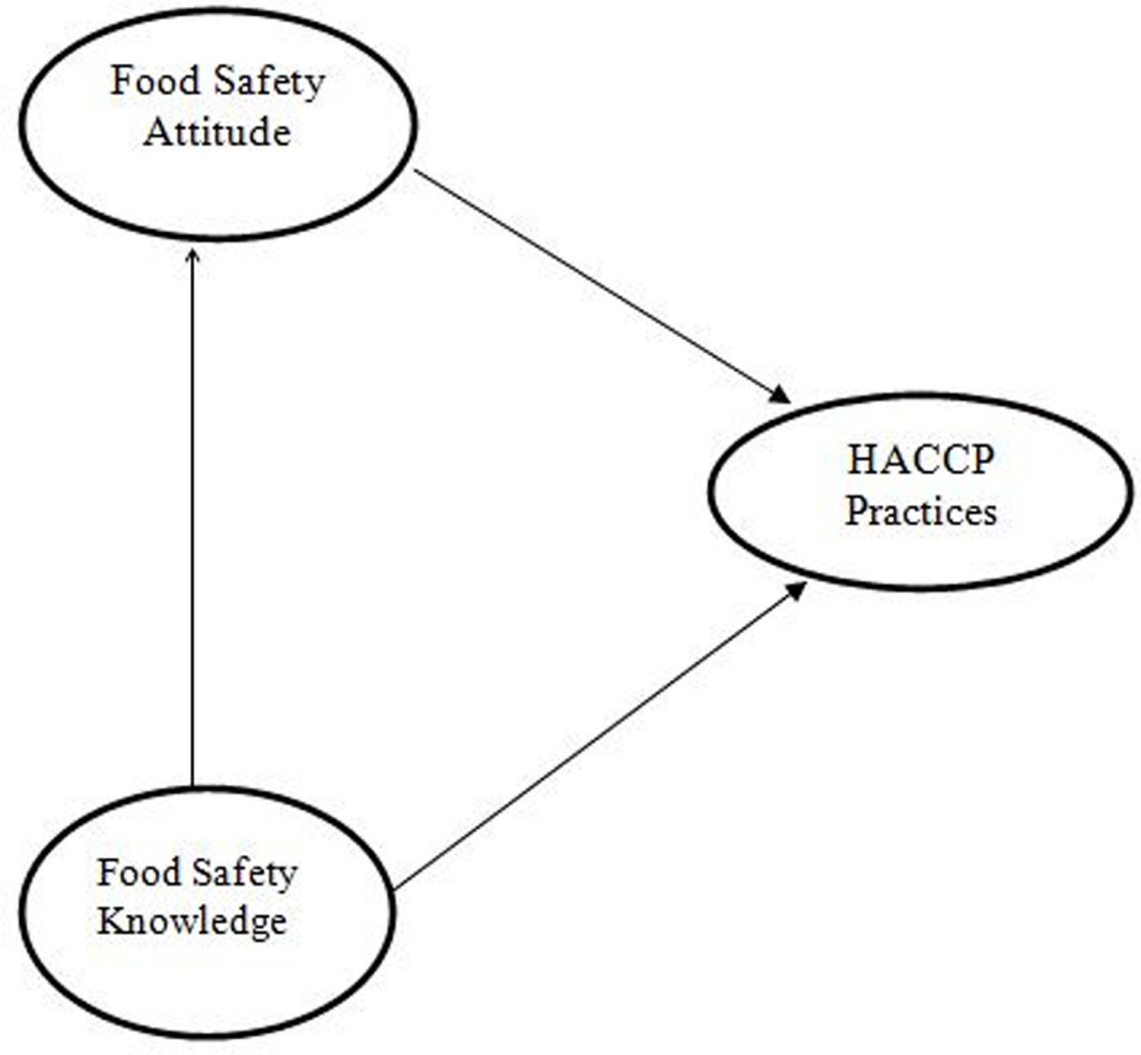
Model development.

As food contamination and adulteration currently have become serious public health concerns in Bangladesh, the fight against them has become tough. Therefore, the country’s government established the Bangladesh Food Safety Authority (BFSA) in 2014. But now, BSFA has faced numerous problems with a regular monitoring system to assess food contamination and its impact on public health, which is now a matter of concern. Recently, BFSA has adopted a restaurant grading system to help consumers. The BFSA provides colored stickers namely green, blue, yellow, and orange to the restaurants based on the food quality, purity, cleanliness, waiters’ health, decoration, and kitchen conditions [7]. Food contamination could occur and contribute to foodborne diseases by employees if they fail to adhere to proper food handling practices in their respective places [8]. The most common foodborne disease in Bangladesh is diarrhea, which can cause death at some point. Data from the International Centre for Diarrheal Disease Research, Bangladesh (ICDDR, B) show that an average of 501 people are admitted to hospitals because of diarrhea every day [7]. The common causes of food poisoning are cross-contamination, insufficient heating, keeping food at room temperature for extended periods, infected food handlers, using contaminated materials, and inadequately cleaned equipment [9, 10]. HACCP is an internationally recognized system managed by the international food safety community to reduce the risk of food safety hazards [11]. HACCP is a system that identifies, evaluates, and controls hazards that are significant for food safety, and it is based on prevention rather than mainly relying on end-product testing. HACCP allows a detailed examination of every process to identify the potential hazards and determine whether they can be controlled [12]. Running the HACCP system in restaurants, whether small or large, is not an easy task due to the lack of knowledge, attitudes, focus, trained human resources, technological equipment, and adequate finances [13]. Food handlers need the skills and knowledge to handle foods safely. Some studies show that training improves knowledge, but a relationship that exists between knowledge, attitude, and practice is necessary, as knowledge alone is not sufficient to cause a change in practices [14, 15]; however, other reports demonstrate that training can improve food handling practices [16, 17]. It is also suggested that the individual’s attitude and behavior are not related to knowledge [18]. Nevertheless, the study of Hosen and Afrose in Bangladesh on microbial quality of restaurant food revealed that microbial contamination occurred due to a lack of knowledge, practices, and information about the route of microbial contaminations, proper hygiene, and sanitation practice [5]. There is also a lack of information regarding Knowledge, Attitude, and Practice (KAP) among the restaurant employees. Recently, the government of Bangladesh has gained certification for large-scale food service providers. Hence, it is essential to understand the present status of KAP among the restaurant employees to offer suggestions to foodservice industry executives and government officials or regulatory authorities on how to improve the enforcement of HACCP-based food safety systems. Concerning the aforementioned lacunae, this study aims to investigate and evaluate the relationships among knowledge, attitude, and HACCP practices in restaurant employees in Bangladesh. Thus, we expected to establish a liaison between the issues affecting food safety knowledge, attitude, and HACCP practices using SEM, which is usually employed as the confirmatory technique for the determination of the validity of the method and seeks to explain the relationships among multiple variables. Fig 1 shows the hypothesized interrelationships between these factors in the present study. Moreover, as food safety is one of the current key issues in Bangladesh, it is also expected that the findings of this study will contribute significantly toward developing a restaurant HACCP plan as well as a policy to supply safe food to the country.

## Methods

### Study design and participants

In this study, the respondents were selected using quota sampling. The study was conducted among the part-time and full-time restaurant employees in Sylhet city situated in the northeastern part of Bangladesh, where a large number of tourists visit every year [19, 20]. The draft versions of the original English questionnaires were translated into Bangla, the national language of Bangladesh. A pilot study was conducted in March 2019 among fifty participants to assess the clarity of the questions, identify response options, and gauge the likely interview length. We used pilot test results, item analysis, factor analysis, and other recommendations to revise the questionnaire by consulting with experts and volunteers following a series of meetings. From 120 roadside general restaurants, a total of 360 employees, among which 92.67% were involved in the preparation, cleaning and washing dishes, serving, participated in this study from September to October 2019. The restaurants were selected considering that they are popular and affordable for all strata of society. Respondents were interviewed on a one-to-one and a face-to-face basis and were given enough time to answer the questionnaire. All steps of the study followed the guidelines of the Strengthening the Reporting of Observational Studies in Epidemiology (STROBE) [21].

### Measures

The questionnaire included four sections. Section one encompassed the participants’ demographic information such as gender, age, work experience (years), education, marital status, the employees’ department, and supervision of the participant. Section two included 32 yes-no questions to assess food safety knowledge. Questions were divided into three categories: food poisoning, GHP standards, and HACCP standards. For section two, each correct answer was given 1 point, and the wrong answer scored 0 points. The reliability index (KR-20) was 0.60 for food safety knowledge.

Sections three and four addressed the food safety attitudes and HACCP practices, respectively. Data were collected from a series of items in which scores were calculated using a 5-point Likert-type scale ranging from 1 (strongly agree) to 5 (strongly disagree). We addressed our initial questions in section three by performing item and factor analysis. Item analysis weighed performance test items based on the assumption that the overall test feature reflects the attributes of its factor’s items. The item analysis indexes such as mean, standard deviation, corrected item-total correlation, skewness, and item discrimination were evaluated. Exploratory Factor Analysis (EFA) deals with the relationships among the observed values in terms of their basic factors. In this study, we used the varimax rotation EFA method, with a minimum eigenvalue of 1.0 used for factor extraction.

The food safety attitude questionnaire included 14 questions across two paradigms, such as self-improvement (6 items) and food safety concern (8 items), and Cronbach’s α for the two constructs ranged from 0.710 to 0.83, indicating good reliability. In the HACCP practices section, we finalized five questions following the item analysis. Cronbach’s α for all scales was 0.816.

## Declarations

### Ethics approval and consent to participate

The data collection procedure for this was carried out with the approval of the Office of Research Compliance of Shahjalal University of Science and Technology (SUST), Bangladesh review board (No. FET/M/19/014). Before the interview, written informed consent was obtained from individual respondents, and this was followed by an oral and written explanation given by the interviewers. The respondents were informed of the voluntary nature of the survey, the potential risks involved in participation, the purpose of the gathered data (assessment of health needs and planning food services), and the confidentiality of the results of the individual interview.

### Consent for publication

Before interviewing the survey team orally explains the aim of the study and no personal information will be included during the publication.

### Statistical analysis

We performed the analysis as per the proposed properties like SEM and a LISREL (Linear Structural Relations) procedure followed by correlation matrices and standard deviations to test the hypothesized model [22]. All routes in the projected model were found, reflecting a causal link among the variables and latent constructs. To measure the model fit, we performed several measures. As the conventional χ^2^ fit test is used to test accurate fit, we refused it due to its inapplicability for this study. We chose to use χ^2^/df [23], the Root Mean Square Error of Approximation (RMSEA) [24, 25], the comparative fit index (CFI) [26], the Tucker-Lewis index (TLI) [26], Standardized Root Mean Square Residual (SRMR), and the Coefficient of Determination (CD) instead [27].

## Results

### Descriptive studies

All the participants were male and aged 20–39 years. More than half of the respondents (52.78%) were married, and most (75%) had 1–6 years of work experience. Most (80.56%) of participants were high school or college graduates, and (75%) of employees were not supervised. About 75% of respondents were serving the food (Table 1).

**Table 1:** Participant’s demographic characteristics.

Variable	Item	Number	Percent (%)
Gender	Male	360	100
	Female	0	0
Age	Under 19	0	0
	20–24	100	27.78
	25–29	120	33.33
	30–34	110	30.56
	35–39	30	8.33
	40–44	0	0
	45–49	0	0
	Over50	0	0
Nationality	Bangladeshi	360	0
	Others	0	0
Marital Status	Single	170	47.22
	Married	190	52.78
	Divorce	0	0
Work experience(years)	Under 1	0	0
	1–3	140	38.89
	4–6	130	36.11
	7–9	60	16.67
	10–12	10	2.78
	13–15	10	2.78
	Over 16	10	2.78
Education	No formal education	0	0
	Primary school	70	19.44
	High school	200	55.56
	College	70	19.44
	University	20	5.56
Department	Cooking	50	13.89
	Cleaning and washing dishes	10	2.78
	Serving food	270	75.00
	Preparation of food ingredients	0	0
	Others	30	8.33
Does a supervisor check your work?	Yes	90	25.00
	No	270	75.00

### Descriptive statistics of measurement items

We considered food poisoning, GHP, and HACCP practices in the food safety knowledge section (Table 2). The results demonstrated that the average score for this section was 53.32%. The GHP was comprised of the highest average score (64.53%), and the HACCP encompassed the lowest average score (37.65%). Around 57.78% of the respondents correctly related to the safety question. The items “have you heard the name Hazard Analysis Critical Control Point (HACCP)” and “do you familiar with the principle of HACCP” earned a score of 11 and 44.44.

**Table 2:** Percentage of correct responses on food safety knowledge (N = 360).

Construct	Item	Correct (%)
Food poisoning	Food poisoning occurs if it is not cooked or reheated thoroughly	72.22
	Food poisoning occurs if it is not stored correctly–for example, it has not been frozen or chilled	69.44
	Food poisoning occurs if it is left out for too long	69.44
	Food poisoning occurs if it is handled by someone who is ill or has not washed their hands	63.89
	Food poisoning occurs due to cross-contamination.	50.00
	Food poisoning occurs more frequently in summer than winter	63.89
	*Clostridium botulinum* is caused by improper vacuum and packing.	47.22
	*Bacillus cereus* is caused by the improper cooking and cooling of rice	41.67
	*Vibrio parahaemolyticus* is found in waters where shellfish are harvested	50.00
	People with infected cuts should cover their wounds and avoid touching food	50.00
Total		57.78
Good hygiene practices	Food handlers must wear clean and appropriate uniforms and follow dress codes, including removing jewelry from hands	75.00
	Clean the fridge and freezer regularly	52.78
	Steam clean The Cooker Hood	27.78
	Nails must be trimmed and clean, without nail polish	75.00
	Staff must wear hats, covering all the hair	80.56
	A hat must be put on before entering the kitchen so as not to transfer microorganisms on food by coming or fixing hair in the kitchen	75.00
	Rubber gloves must be worn during dishwashing	69.44
	Smoking should be forbidden in the kitchen and adjacent areas.	91.67
	Use hot dry sterilization at 110 C for over 30 min to clean utensils	47.22
	Wash knives and cutting boards only at the end of each business day	80.56
	Wear perfume or aftershave while preparing food	77.78
	Packaged raw ingredients can be placed directly on the floor	44.44
	The holding temperature is above 50°C. and the refrigerator temperature is below 10°C	41.67
Total		64.53
HACCP	You heard the name “Hazard analysis critical control points	44.44
	“HACCP” the best method to control food safety in the world	0.00
	You familiar with the principles of HACCP	11.11
	HACCP emphasizes prevention rather than inspection	0.00
	HACCP is a method to manage critical questions in advance to achieve prevention objectives	50.00
	HACCP addresses final product quality, not preparation procedures	52.78
	HACCP effectively uses human and material resources and may decrease food processing costs	63.89
	Microbiological hazards cannot be included in HACCP	52.78
	It is essential to keep track of and to record every step of food production in the HACCP system	63.89
Total		37.65
Average Percentage of GHP, Food Poisoning, HACCP		53.32

Table 3 represents the score of food safety attitude. We considered self-improvement and food safety concerns in this section. The self-improvement construct earned the highest average score (2.78), followed by the food safety concern (2.71). Table 4 represents that the mean score for HACCP practices was 3.09.

**Table 3:** Mean values of participants’ food safety attitude scores.

Construct	Item	Mean	S.D.
Self-improvement	You read more journals about food safety to increase your food sanitation knowledge	2.75	1.23
	You think attending a sanitation seminar would change your sanitation behavior	2.14	1.22
	You think attending a sanitation seminar would increase your sanitation knowledge and ideas	2.06	.95
	You think Learning more about food safety through training courses is important to you	3.06	1.31
	You think you do not need to attend a food safety seminar because you think you have sufficient knowledge about food safety	3.67	1.26
	You attend a cooking or service competition to increase your professional knowledge	3.03	1.32
Total		2.78	0.53
Food safety concern	Government is responsible for preventing food poisoning	2.5	1.13
	Consumers are responsible for preventing food poisoning	2.39	.96
	Maintaining a clean cooking environment is a good way to control food safety	2.36	1.13
	Self-checking food safety is important to restaurants and institutions	2.58	1.23
	Food safety is more important than taste	2.53	1.18
	Food safety knowledge is important to ensure that food is prepared in a safe manner	2.78	1.22
	Food poisoning is not a serious matter	3.72	1.23
Total		2.71	0.55

**Table 4:** HACCP Practice for the employees.

Item	Mean	S.D.
I have a plan to achieve my HACCP goal.	3.17	1.18
I respect HACCP plan goals	3.72	1.03
I usually follow the HACCP plan to maintain food safety.	2.56	1.05
Knowledge of food sanitation helps to perform my job correctly	3.11	1.28
I try hard to maintain food sanitation standards	2.86	1.25
Total	3.09	0.79

### Structural model

The relationships between food safety knowledge, attitude, and HACCP practices are presented in Table 5. The results indicate that food safety knowledge was negatively correlated with attitude and HACCP practices; however, the attitude was positively correlated with HACCP practices, and there was a significant positive correlation (*p* < 0.05) between HACCP practices and shelf improvement. The knowledge toward food poisoning and GHP practices was significantly correlated (*p* < 0.05), in contrast, there was no significant relationship between food poisoning and HACCP practices. Lastly, we found self-improvement and food safety concerns are negatively correlated with food poisoning, GHP, and HACCP practice.

**Table 5:** Relationships among participant food safety knowledge, attitude, and HACCP practices of the participants (N = 360).

	Knowledge				Attitude	
Construct	Food poisoning	GHP	HACCP	Self-improvement	Food safety concern	HACCP practices
**Food Safety knowledge**						
Food poisoning	1.0000					
GHP	0.5518*	1.0000				
HACCP	0.2899	0.2297	1.0000			
**Food safety attitude**						
Self-improvement	-0.2815	-0.0762	-0.2521	1.0000		
Food safety concern	-0.2536	-0.2970	-0.1280	0.3155	1.0000	
**HACCP practices**	-0.2322	-0.2408	-0.207	0.5388*	0.2041	1.0000

*Correlation is significant at *p* < 0.05 level (2-tailed).

**Fig 2:**
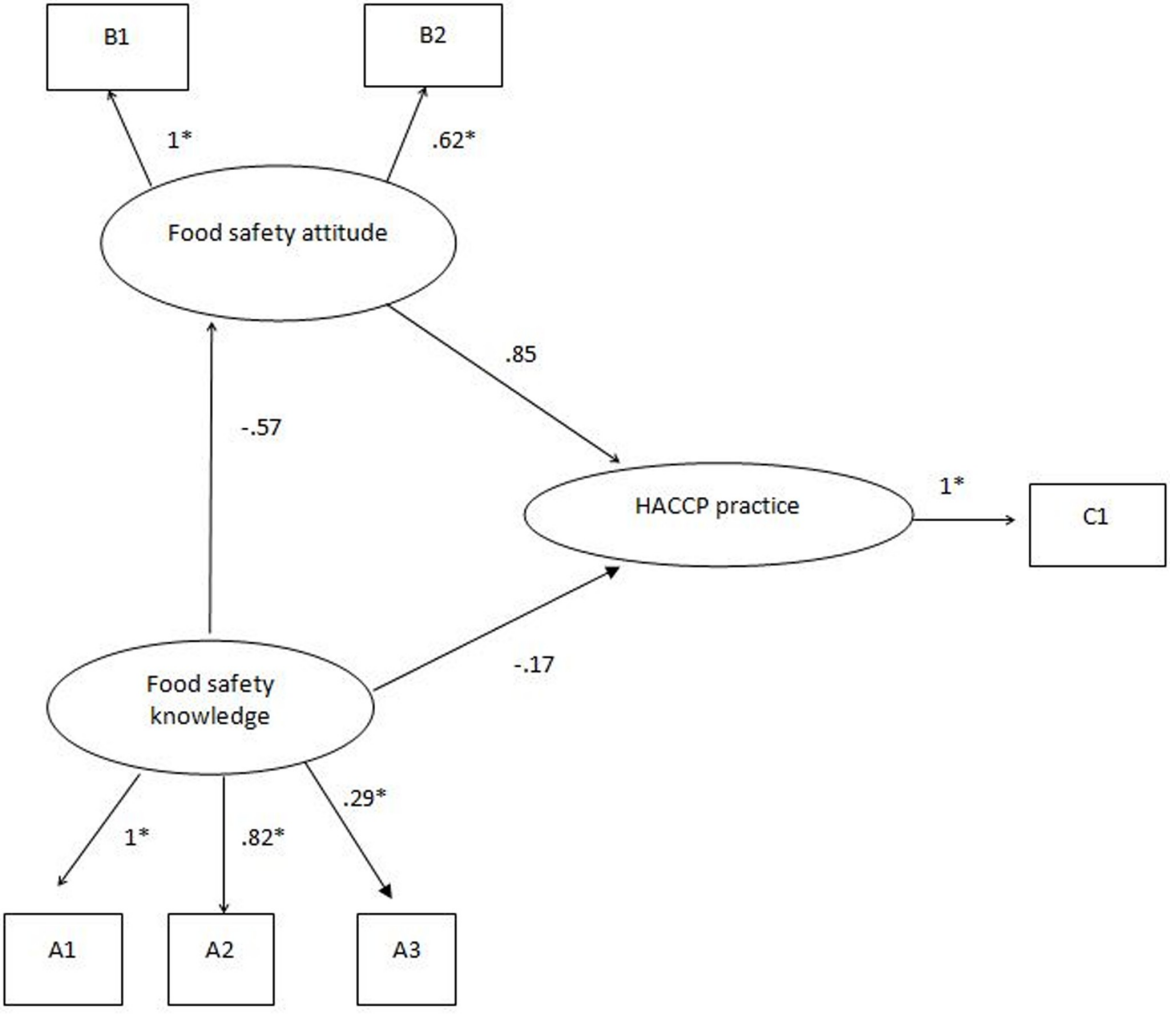
Model of food safety knowledge, attitude, and HACCP practice. A1: Food poisoning knowledge; A2: GHP knowledge; A3: HACCP knowledge; B1: Self-improvement attitude; B2: Food safety concern; C1: HACCP implementation.

We used structural equation modeling to illustrate the relationship among food safety knowledge, attitude, and HACCP practices, as presented in Fig 2. Analytical results indicated a negative relationship between food safety knowledge and attitude and between food safety knowledge and HACCP practices. The SEM was used to investigate the fit indices and variance-explained estimates. Table 6 presents a variety of indices used to evaluate the model’s overall value, indicating a good model fit.

**Table 6:** Goodness-of-fit indices for the measurement model.

Fit indices	X^2^(Chi-square)	DF	X^2^/DF	RMSEA	CFI	TLI	SRMR	CD
Measurement	6.196	7	0.885	0.00	1.00	1.06	0.06	0.78
Accepted value			<3^a^	<0.08^b^	≥0.90^c^	>0.90^d^	<0.08^e^	0–1^f^

Chi-square/DF (X^2^/DF), ^a^ [39]

DF = Degree of freedom

RMSEA = Root mean square error of approximation. ^b^ [25]

CFI = Comparative fit index. ^c^ [40]

TLI = Tucker-Lewis index. ^d^ [26]

SRMR = Standardized root mean squared residual. ^e^ [41]

CD = Coefficient of determination. ^f^ [27].

The model for food safety knowledge, attitude, and HACCP practices were verified by SEM, and the goodness of fit of the model was evaluated. In this study, the χ^2^ for the measurement of model fit was 6.196 with 7 degrees of freedom (df). Theoretically, when the assumption was made, χ^2^ was insignificant, and an χ^2^/df ratio of < 5 was acceptable, but the ratio of < 3 was preferred [23]. The χ^2^/df ratio for the measurement model in this study was 0.89, indicating a good fit for the sample [23]. Our model was also evidently a good fit, according to the RMSEA, the Comparative Fit Index (CFI), Tucker-Lewis Index (TLI), SRMR, and the (CD) values. Our results showed that RMSEA and SRMR were 0.0001 and 0.06, respectively, and in both cases, the value was < 0.08 [24–26]. Moreover, CFI and TLI showed values of 1 and 1.06, respectively, indicating that both cases were good CFI (their values were greater than 0.90) [26]. A value close to 1 indicates a good fit according to CD, and in our study, it appeared as 0.78 [27]. As shown in Table 6, the requirements were fulfilled; thus, the hypothesized model in this study was a good fit and acceptable.

## Discussion

Food safety is not salient to achieve the Sustainable Development Goals (SDGs) by 2030 but may become more important to ensure good health and wellbeing [28]. This research provides information and an outline of many complex questions about the knowledge, attitudes, and practices of the employees working in restaurants in Bangladesh. This study revealed that food handlers had above average knowledge regarding food poisoning and good hygiene practices, but the knowledge of HACCP practices was below the average. There is a lack of information regarding the HACCP practices in Bangladesh, although they are important for industry. The HACCP system provides a high degree of food safety assurance, many enormities by the traditional approaches, over that exist for foodservice operations [29]. It appears that employees had almost zero knowledge about the principles of HACCP. Therefore, intensified efforts need to be made to implement the HACCP system in foodservice operations.

Although training may demonstrate increased knowledge, it does not always result in a change in HACCP practices and attitude. Restaurants must decide which food safety system best suits in their specific situation and how the system should be implemented. Besides, GHP implementation is difficult for restaurants, as high-quality food is necessary to earn and maintain consumer trust and loyalty. However, although in this study, the level of GHP knowledge concerning food poisoning and HACCP knowledge among restaurant employees is high, it is indeed not at a satisfactory level. Therefore, the country and employers should focus on resolving the prevalent low-level basic GMP, food poisoning, and HACCP knowledge. Therefore, periodic evaluation of food handlers’ knowledge and the food safety training materials should be made. Many studies revealed that introducing the HACCP system in restaurants improves the quality of food [30–32].

Our study found positive correlations between self-improvement and food safety concerns and between HACCP practices and attitude. These results are similar to an earlier study by Ko [9]. The score of HACCP practices was only 0.79. Many researchers have concluded that training is important to improve standards of food hygiene, positively change the behavior of employees, and prevent foodborne diseases [17, 33, 34]. A combination of positive knowledge and attitude is necessary to improve food handling safety [35].

Fig 2 demonstrates the standardized path coefficients for the structural model under investigation, determining the strength of the direct relationship between constructs. Our results showed a negative relationship between food safety knowledge (food poisoning, GHP, and HACCP) and food safety attitude (self-improvement and safety concern), implying that though the respondents have the knowledge related to food safety, they do not apply it to change their behavior (Fig 2). This situation can improve through legislative governance of food safety guidelines by the local governments like the city corporation as well as the BFSA. In Bangladesh, there is no well-established food safety governance and monitoring system for restaurant foods. Besides, employee training may increase food safety knowledge and hygienic awareness and potentially improve the HACCP practices.

All restaurant managers, supervisors, and operators have important roles in the implementation of the HACCP system. Fletcher et al. reported that the staff working in large hotels should have sufficient knowledge about HACCP plans, HACCP principles, and related standard operating procedures [36]. Indeed, to improve knowledge transfer, feedback, self-control, and individual commitment to HACCP, training program along with legislation play essential roles [37, 38]. The results of this study were in agreement with the findings obtained by Lim, Chye [39], in which knowledge had a negative relationship with both attitude and practices.

The present study had some limitations. For example, we were not able to interview all restaurants of the Sylhet division and relied on the answers of the participants and used quota sampling methods to select the respondents. The participants may answer some questions correctly, which may or may not truly indicate what they do and/or the actual scenario. Moreover, there may be other relevant confounders we might have overlooked.

## Conclusion

This study demonstrates that food safety knowledge had a negative relationship with both attitude and HACCP practices; however, the attitude had a positive relationship with HACCP practices. Moreover, this study revealed that if an individual has a fair level of food safety knowledge; it does not necessarily turn into a positive attitude or a tendency to practice HACCP. Therefore, a piece of legislation to follow the food safety guidelines is of great importance, along with the establishment of strong monitoring and a frequent training program for the country.

## Supporting information

S1 Questionnaire Understanding the relationship among food safety knowledge, attitude, and self-reported HACCP practices in restaurant employees in Bangladesh. (DOCX)
