# Supplementary material for: Structural modeling to understand the relationship among food safety knowledge, attitude, and self-reported HACCP practices in restaurant employees in Bangladesh
Source: PLOS Glob Public Health. 2022 May 6;2(5):e0000103. doi: 10.1371/journal.pgph.0000103 (PMC10021393; doi:10.1371/journal.pgph.0000103)
Supplement: S1 Questionnaire — (DOCX) [file pgph.0000103.s001.docx]

# Appendix

**Ethical Statement**

The purpose of this study is to know about the Knowledge of restaurant employees about food safety and their attitudes and self reported HACCP practices in restaurants in sylhet . This study is being conducted through the students of Shahjalal university of science & technology, sylhet. This questionnaire asks about Food safety knowledge, Food safety attitude, HACCP practice for the employees. Your response will be anonymous and will never be linked to you personally. Your participation is entirely voluntary. If there are any items you feel uncomfortable answering, please skip those items. Thank you for your co-operation.

Signature:

**Participant demographic characteristics**.

1. Name:

2. Gender:

- Male
- Female

3. Age:

- Under 19
- 20-24
- 25-29
- 30-34
- 35-39
- 40-44
- 45-49
- Over 50

4.Nationality

- Bangladeshi
- Or others

5.Marital status

- Single
- Married
- Divorce

6.Work experience (years)

- Under 1
- 1-3
- 4-6
- 7-9
- 10-12
- 13-15
- Over 16

7. Education

- No formal education
- Primary school
- High school
- College
- University

8. Department

- Cooking
- Cleaning and washing dishes
- Serving food
- Preparation of food ingredients
- Others

9. Does a supervisor check your work?

a)Yes b)No

**Food safety knowledge**

**Food poisoning:**

10. Does food poisoning occurs if it isn't cooked or reheated thoroughly?

a)Yes b)No

11. Does food poisoning occurs if it isn't stored correctly – for example, it's not been frozen or chilled?

a)Yes b)No

12. Does food poisoning occurs if it is left out for too long?

a)Yes b)No

13. Does food poisoning occurs if it is handled by someone who's ill or hasn't washed their hands?

a) Yes b) No

14. Does food poisoning occurs due to cross contamination?

a)Yes b)No

15. Does food poisoning occurs more frequently in summer than winter?

a) Yes b)No

16. Does clostridium botulinum is caused by improper vacuum and can packing. Without medical treatment, death is likely?

a)Yes b)No

17. Does Bacillus cereus is caused by the improper cooking and cooling of rice?

a)Yes b)No

18.Does Vibrio parahaemolyticus is found in waters where shellfish are harvested?

a)Yes b)No

19. Does people with infected cuts should cover their wounds and avoid touching food?

a)Yes b)No

**Good hygiene practices**

20. Does food handlers must wear clean and appropriate uniforms and follow dress codes, including removing jewelry from hands?

a)Yes b)No

1. Does it require to clean The Fridge And Freezer regularly?

a)Yes b)No

1. Does it require to Steam clean The Cooker Hood?

a)Yes b)No

1. Nails must be trimmed and clean, without nail polish?

a)Yes b)No

1. Staff must wear hats, covering all the hair?

a)Yes b)No

1. Hat must be put on before entering the kitchen so as not to transfer microorganisms on food by coming or fixing hair in the kitchen?

a)Yes b)No

1. Rubber gloves must be worn during dish washing?

a)Yes b)No

1. Smoking should be forbidden in the kitchen and adjacent areas?

a)Yes b)No

1. Does use dry hot sterilization at 110^0^ C for over 30 min to clean utensils?

a)Yes b)No

1. Does wash knives and cutting boards only at the end of each business day?

a)Yes b)No

1. Do you wear perfume or aftershave during preparing food?

a)Yes b)No

1. Does packaged raw ingredients can be placed directly on the floor?

a)Yes b)No

1. Does holding temperature is above 50 ^o^C. and refrigerator temperature is below 10 ^0^c?

a)Yes b)No

**HACCP**

1. Have you heard the name “Hazard analysis critical control points”?

a)Yes b)No

1. Is “HACCP” the best method to control food safety in the world?

a)Yes b)No

1. Are you familiar with the principles of HACCP?

a)Yes b)No

1. HACCP emphasizes prevention rather than inspection?

a)Yes b)No

1. HACCP is a method to manage critical questions in advance to achieve prevention objectives?

a)Yes b)No

1. HACCP addresses final product quality, not preparation procedures?

a)Yes b)No

1. HACCP effectively uses human and material resources and may decrease

food processing costs?

a)Yes b)No

1. Microbiological hazards cannot be included in HACCP ?

a)Yes b)No

1. It is essential to keep track of and to record every step of food production in HACCP system?

a)Yes b)No

**Food safety attitude**

**Self improvement**

1. Do you read more journals about food safety in order to increase your food sanitation knowledge ?

a)Strongly agree b)Agree c)Neutral d)Disagree e)Strongly disagree

1. Do you think attending a sanitation seminar would change your sanitation behavior?

a)Strongly agree b)Agree c)Neutral d)Disagree e)Strongly disagree

1. Do you think attending a sanitation seminar would increase your sanitation knowledge and ideas?

a)Strongly agree b)Agree c)Neutral d)Disagree e)Strongly disagree

1. Do you think Learning more about food safety through training courses is important to you?

a)Strongly agree b)Agree c)Neutral d)Disagree e)Strongly disagree

1. Do you think you do not need to attend food safety seminar because you think you have sufficient knowledge about food safety?

a)Strongly agree b)Agree c)Neutral d)Disagree e)Strongly disagree

1. Do you attend a cooking or service competition to increase your professional knowledge?

a)Strongly agree b)Agree c)Neutral d)Disagree e)Strongly disagree

**Food safety concern**

1. Does Food handlers are responsible to prevent food poisoning.?

a)Strongly agree b)Agree c)Neutral d)Disagree e)Strongly disagree

1. Government is responsible to prevent food poisoning ?

a)Strongly agree b)Agree c)Neutral d)Disagree e)Strongly disagree

1. Consumers are responsible to prevent food poisoning ?

a) Strongly agree b)Agree c)Neutral d)Disagree e)Strongly disagree

1. Is Maintaining a clean cooking environment is a good way to control food safety ?

a)Strongly agree b)Agree c)Neutral d)Disagree e)Strongly disagree

1. Is Self-checking food safety is important to restaurants and institutions ?

a)Strongly agree b)Agree c)Neutral d)Disagree e)Strongly disagree

1. Is Food safety is more important than taste?

a)Strongly agree b)Agree c)Neutral d)Disagree e)Strongly disagree

1. Food safety knowledge is important to ensure food is prepared in a safe manner?

a)Strongly agree b)Agree c)Neutral d)Disagree e)Strongly disagree

1. Food poisoning is not a serious matter?

a)Strongly agree b)Agree c)Neutral d)Disagree e)Strongly disagree

**HACCP practice for the employees**.

1. I have a plan to achieve my HACCP goal?

a)Strongly agree b)Agree c)Neutral d)Disagree e)Strongly disagree

1. I respect HACCP plan goals?

a)Strongly agree b)Agree c)Neutral d)Disagree e)Strongly disagree

1. I usually follow the HACCP plan to maintain food safety?

a)Strongly agree b)Agree c)Neutral d)Disagree e)Strongly disagree

1. Knowledge of food sanitation helps to perform my job correctly?

a)Strongly agree b)Agree c)Neutral d)Disagree e)Strongly disagree

1. I try hard to maintain food sanitation standards?

a)Strongly agree b)Agree c)Neutral d)Disagree e)Strongly disagree
